# Efficacy and Safety Results With Rilzabrutinib, an Oral Bruton Tyrosine Kinase Inhibitor, in Patients With Immune Thrombocytopenia: Phase 2 Part B Study

**DOI:** 10.1002/ajh.27539

**Published:** 2025-01-22

**Authors:** Nichola Cooper, A. J. Gerard Jansen, Robert Bird, Jiří Mayer, Michelle Sholzberg, Michael D. Tarantino, Mamta Garg, Paula F. Ypma, Vickie McDonald, Charles Percy, Milan Košťál, Isaac Goncalves, Lachezar H. Bogdanov, Terry B. Gernsheimer, Remco Diab, Mengjie Yao, Ahmed Daak, David J. Kuter

**Affiliations:** ^1^ Department Immunology and Inflammation, Imperial College Hammersmith Hospital London UK; ^2^ Erasmus MC University Medical Center Rotterdam The Netherlands; ^3^ Princess Alexandra Hospital Woolloongabba Australia; ^4^ Masaryk University Hospital Brno Czech Republic; ^5^ St. Michael's Hospital, Li Ka Shing Knowledge Institute University of Toronto Toronto Ontario Canada; ^6^ The Bleeding and Clotting Disorders Institute University of Illinois College of Medicine‐Peoria Peoria Illinois USA; ^7^ Leicester Royal Infirmary Leicester UK; ^8^ Department of Hematology HagaZiekenhuis, Den Haag The Netherlands; ^9^ Barts Health NHS Trust The Royal London Hospital London UK; ^10^ Queen Elizabeth Hospital Birmingham UK; ^11^ Fourth Department of Internal Medicine and Hematology, Faculty of Medicine University Hospital of Hradec Králové Hradec Králové Czech Republic; ^12^ Royal Melbourne Hospital and Peter MacCallum Cancer Centre Parkville Australia; ^13^ Clinic of Hematology University Hospital Pleven Bulgaria; ^14^ University of Washington and Fred Hutchinson Cancer Center Seattle Washington USA; ^15^ Sanofi Rotkreuz Zug Switzerland; ^16^ Sanofi Bridgewater New Jersey USA; ^17^ Sanofi Cambridge Massachusetts USA; ^18^ Hematology Division, Massachusetts General Hospital Harvard Medical School Boston Massachusetts USA

**Keywords:** adults, immune thrombocytopenia, platelets, quality of life, response

## Abstract

Current treatments for persistent or chronic immune thrombocytopenia (ITP) are limited by inadequate response, toxicity, and impaired quality of life. The Bruton tyrosine kinase inhibitor rilzabrutinib was evaluated to further characterize safety and durability of platelet response. LUNA2 Part B is a multicenter, phase 1/2 study in adults with ITP (≥ 3 months duration, platelet count < 30 × 10^9^/L) who failed ≥ 1 ITP therapy (NCT03395210, EudraCT 2017–004012‐19). Oral rilzabrutinib 400 mg bid was given over 24 weeks, with optional long‐term extension (LTE). Primary endpoints were safety and platelet counts ≥ 50 × 10^9^/L on ≥ 8 of the last 12 weeks of main treatment without rescue medication. From 22 March2018 to 31 January2023, 26 patients were enrolled. Patients had baseline median platelet count 13 × 10^9^/L, ITP duration 10.3 years, and six prior ITP therapies (46% splenectomized). Nine (35%) patients achieved the primary endpoint. Platelet counts ≥ 50 × 10^9^/L or ≥ 30 × 10^9^/L and doubling from baseline without rescue therapy were sustained for a mean 9.3 weeks. 11 (42%) LTE‐eligible patients were ongoing with median LTE platelet > 80 × 10^9^/L. Three (12%) patients received rescue medication during main treatment, none in LTE. Clinically meaningful improvements were observed in fatigue and women's health. With a median treatment duration of 167 days (main treatment), 16 (62%) patients had ≥ 1 treatment‐related adverse event (AE), mainly grade 1, including diarrhea (35%), headache (23%), and nausea (15%). There was no treatment‐related grade ≥ 2 bleeding/thrombotic events/infections, serious AE, or death. Rilzabrutinib continues to demonstrate durable platelet responses with favorable safety profile in previously treated ITP patients.

**Trial Registration:** NCT03395210, EudraCT 2017‐004012‐19

## Graphical abstract

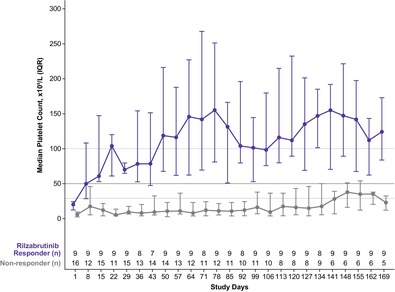

## 1 Introduction

Immune thrombocytopenia (ITP) is an autoimmune disease characterized by immune‐mediated destruction and impaired production of platelets, resulting in isolated thrombocytopenia (i.e., platelets < 100 × 10^9^/L) that contributes to elevated risks for bleeding and reduced quality of life (QOL) [1, 2, 3, 4]. Standard first‐line ITP therapy for adults includes corticosteroids, intravenous immunoglobulin (IVIg), or anti‐D aiming to rapidly increase platelet counts and minimize bleeding [2, 5]. Despite initial responses, durable remission is uncommon and patients frequently relapse, requiring consideration of alternate treatment options, such as thrombopoietin‐receptor agonists (TPO‐RA), rituximab, fostamatinib, other immunosuppressive agents, and/or splenectomy [2, 5]. Unmet needs for patients with ITP continue to focus on identifying effective treatment to ensure sustained platelet count increases and prolonged remission, as well as reducing long‐term toxicity and improving patient QOL.

The broad expression of Bruton tyrosine kinase (BTK) in immune cells and role in B‐cell maturation, antibody production, and Fc receptor‐mediated signaling pathways make it a therapeutic target for many immune‐mediated disorders [6, 7]. Early clinical evidence identified a potential role in patients with autoimmune cytopenias (e.g., ITP) secondary to chronic lymphocytic leukemia, who showed increased platelet counts and fewer episodes of autoimmune‐based cytopenic events following BTK inhibitor treatment [8, 9]. Rilzabrutinib is a potent oral, reversible BTK inhibitor designed to treat autoimmune disorders based on its potential to modulate multiple immunological pathways [10, 11]. Preclinical mechanisms include inhibition of B‐cell activation, interruption of antibody‐coated cell phagocytosis by Fcγ receptor signaling in spleen and liver, and induction of sustained anti‐inflammatory effects [10]. A phase 1 study in healthy volunteers showed that rilzabrutinib was well‐tolerated with rapid and sustained high levels of BTK occupancy [12]. The phase 1/2, part A, dose‐escalation study of rilzabrutinib in previously treated adults with ITP showed rapid and durable platelet responses with a favorable safety profile, including no apparent increase in bleeding risk [13, 14]. Part B enrolled a completely separate and unique group of ITP patients (different than part A) who failed at least one ITP therapy in addition to corticosteroids or IVIg/anti‐D. In part B, all patients received the selected dose of rilzabrutinib (400 mg bid) with the goal of further characterizing the safety profile and examining the therapeutic effects and durability of platelet response with rilzabrutinib in patients with difficult‐to‐treat ITP.

## 2 Methods

### 2.1 Phase 1/2 Study Design and Participants

Part B of the multicenter, open‐label, phase 1/2 LUNA 2 study evaluated the efficacy and safety of rilzabrutinib 400 mg bid in patients with relapsed ITP (NCT03395210; EudraCT 2017–004012‐19). Patients were enrolled from Australia, Canada, the Czech Republic, the Netherlands, the United Kingdom, and the United States (recruited but not enrolled from Bulgaria).

Eligible patients were aged 18–80 years (18–65 years for the Czech Republic and Norway) with primary or secondary ITP with at least a 3‐month duration. Patients had to have a response (achieved platelet count ≥ 50 × 10^9^/L) to corticosteroids or IVIg/anti‐D that was not sustained and failed at least one other ITP therapy (not corticosteroids or IVIg). Enrolled patients had to have a platelet count < 30 × 10^9^/L on two occasions no less than 7 days apart in the 15 days prior to treatment initiation, and no platelet count > 35 × 10^9^/L on study day 1. Adequate hematologic, hepatic, and renal function were required. Contraceptive use or confirmation of post‐menopausal status were required for females. See appendix for detailed eligibility criteria.

### 2.2 Treatment

Patients received oral rilzabrutinib 400 mg bid over a 24‐week main treatment period, with a 4‐week safety follow‐up for patients who were ineligible for the long‐term extension (LTE) period. Stable concomitant treatment with ≤ 10% dosing variation in corticosteroids and/or TPO‐RA was allowed. Patients completing 24 weeks of rilzabrutinib with platelet counts ≥ 50 × 10^9^/L or ≥ 30 × 10^9^/L and doubling from baseline in ≥ 4 of the last 8 weeks of treatment without rescue medication could continue rilzabrutinib in the LTE until they were no longer responding to rilzabrutinib, rilzabrutinib became commercially available in the patient's country, the program was stopped for safety reasons, or was no longer being developed by the sponsor for ITP.

Patients could receive another ITP therapy (e.g., IVIg, high‐dose steroids, platelet transfusion, or anti‐D immunoglobulin infusion) for a significant safety event to prevent deterioration of their platelet count that in the investigator's opinion was a significant safety risk. Patients receiving rescue therapy could continue rilzabrutinib if no stopping rules were applied and the investigator agreed to continue treatment (see appendix for additional details).

### 2.3 Study Endpoints

The primary efficacy endpoint was the proportion of patients achieving platelet counts ≥ 50 × 10^9^/L on ≥ 8 of the last 12 weeks of the 24‐week treatment period without the use of rescue medication after 10 weeks of active treatment (i.e., durable platelet count response). The primary safety endpoints included the incidence, severity, and relationship of adverse events (AEs) and proportion of patients with grade ≥ 2 bleeding events [15].

Secondary efficacy endpoints included: a. predictive value estimate for achieving the primary endpoint platelet response in the first 8 weeks of treatment, b. the number of weeks with platelet counts ≥ 50 × 10^9^/L or ≥ 30 × 10^9^/L and doubling from baseline in the absence of rescue therapy (platelet counts were censored for 4 weeks after use of rescue therapy, if given), c. proportion of patients achieving ≥ 2 consecutive platelet counts (separated by ≥ 5 days) of ≥ 50 × 10^9^/L and increased ≥ 20 × 10^9^/L from baseline without use of rescue medication in the 4 weeks prior to the latest elevated platelet count, d. number of weeks with platelet counts ≥ 30 × 10^9^/L and doubling from baseline over the 24‐week treatment period (platelet counts were censored for 4 weeks after use of rescue therapy, if given), e. proportion of patients receiving rescue medication, and f. change from baseline in the ITP bleeding scale (IBLS) [16]. Bleeding scores measured by IBLS ranged from 0 to 2, with higher scores indicating marked bleeding. Secondary safety endpoints include the proportion of patients receiving rescue medication and IBLS score at the end of the treatment period.

Exploratory endpoints were the proportion of patients who completed 24 weeks of treatment and demonstrated a platelet response defined as platelet counts ≥ 50 × 10^9^/L at 4 of the last 8 weeks of the active treatment period, proportion of patients on concomitant TPO‐RAs with platelet counts > 250 × 10^9^/L or > 450 × 10^9^/L, time to first platelet count ≥ 50 × 10^9^/L, and percentage of time with platelet counts ≥ 30 × 10^9^/L or ≥ 20 × 10^9^/L above baseline. The effect of rilzabrutinib on markers of hemolysis (haptoglobin), immunoglobulin G (IgG), IgG1, IgG4, IgM, IgE, and thrombopoietin (TPO) levels were assessed. Health‐related QOL (HRQOL) was evaluated with scores from 0 worst to 100 best using the EuroQol‐5 Dimensions five‐Level (EQ‐5D‐5L) + Visual Analog Scale (EQ‐VAS) [17, 18] and ITP Patient Assessment Questionnaire (ITP‐PAQ [19]).

### 2.4 Statistical Analysis

All enrolled patients (signed informed consent and met eligibility criteria) were included in the intent‐to‐treat efficacy analysis population. All enrolled patients who received at least one dose of rilzabrutinib were included in the safety analysis population.

The sample size for part B was designed to estimate the true response rate by enrolling approximately 23 patients. If five responders were observed out of 23 treated patients, the observed response rate was 22% (90% confidence interval [CI], 9%, 40%), with the lower bound of the exact 90% CI excluding 8%.

Demographics and baseline patient characteristics, qualitative efficacy data, and quantitative safety data were summarized using descriptive statistics. Mean changes in platelet counts over time were described graphically. Adverse events were graded in severity by preferred term using the National Cancer Institute Common Terminology Criteria for Adverse Events, version 5.0.

## 3 Results

### 3.1 Patient Disposition

Between March 22, 2018, and January 31, 2023, of 46 screened patients, 26 enrolled, received treatment and were evaluable for efficacy and safety outcomes. Of 26 patients overall, 15 (58%) completed the 24‐week main treatment period, and 11 (42%) met LTE criteria and were ongoing in the LTE (Figure S1). 11 (42%) patients discontinued treatment due to lack of response (*n* = 5), AEs unrelated to treatment (*n* = 3), erroneous enrollment (*n* = 1), non‐compliance (*n* = 1), and lack of response/treatment‐related grade 2 diarrhea (*n* = 1).

### 3.2 Baseline Patient Demographics

Baseline characteristics are summarized in Table 1. Median baseline platelet count was 13 × 10^9^/L (interquartile range [IQR], 6–18 × 10^9^/L). Patients had a median duration of ITP of 10.3 years (IQR, 2.6–18.7) and had received a median of six prior unique ITP therapies (IQR, 4–7), including 12 (46%) patients with prior splenectomy. At baseline, concomitant corticosteroids and/or TPO‐RA (non‐rescue) were given along with rilzabrutinib to 17 (65%) patients (TPO‐RA [*n* = 11], corticosteroids [*n* = 3], and both corticosteroids and TPO‐RA [*n* = 3]).

**TABLE 1:** Baseline patient demographics, disease characteristics, and prior treatment.

Characteristic	Patients (*N* = 26)	
Age, years		
Median (IQR)	57 (32–65)	
Range	20–75	
Sex		
Female	16 (62%)	
Male	10 (38%)	
Platelet count at baseline, × 10^9^/L		
Median (IQR)	13 (6–18)	
Range	2–24	
Duration of ITP^a^, years		
Median (IQR)	10.3 (2.6–18.7)	
Range	0.7–48.2	
Number of unique prior ITP therapies^b^		
Median (IQR)	6 (4–7)	
Range	3–19	
Prior splenectomy		
Yes	12 (46%)	
No	14 (54%)	
Most common prior ITP therapies	Received prior therapy	Prior response^d^
Corticosteroids	26 (100%)	22/26 (85%)
TPO‐RA	22 (85%)	13/22 (59%)
Immunosuppressants^c^	21 (81%)	NR
IVIg	21 (81%)	17/21 (81%)
Rituximab	13 (50%)	2/13 (15%)
Fostamatinib	4 (15%)	NR
Concomitant ITP medication^e^		
TPO‐RA	11 (42%)	
Corticosteroids	3 (12%)	
Corticosteroids + TPO‐RA	3 (12%)	

*Note*: Data are n (%), except where indicated.

Abbreviations: IQR, interquartile range; ITP, immune thrombocytopenia; IVIg, intravenous immunoglobulin; NR, not reported; TPO‐RA, thrombopoietin‐receptor agonist (included eltrombopag or romiplostim).

^a^ Duration of disease is the difference between the date of first rilzabrutinib dose and the date of initial diagnosis.

^b^ Unique ITP therapies and splenectomy may each be counted as one prior ITP therapy.

^c^ Including cyclophosphamide.

^d^ Prior response was defined as having ever achieved platelet count ≥ 50 × 10^9^/L per patient's electronic case report form.

^e^ Stable concomitant corticosteroids and/or TPO‐RA was allowed as defined as having ≤ 10% dosing change within the 2 weeks prior to rilzabrutinib initiation as was allowed per protocol.

### 3.3 Efficacy

**FIGURE 1:**
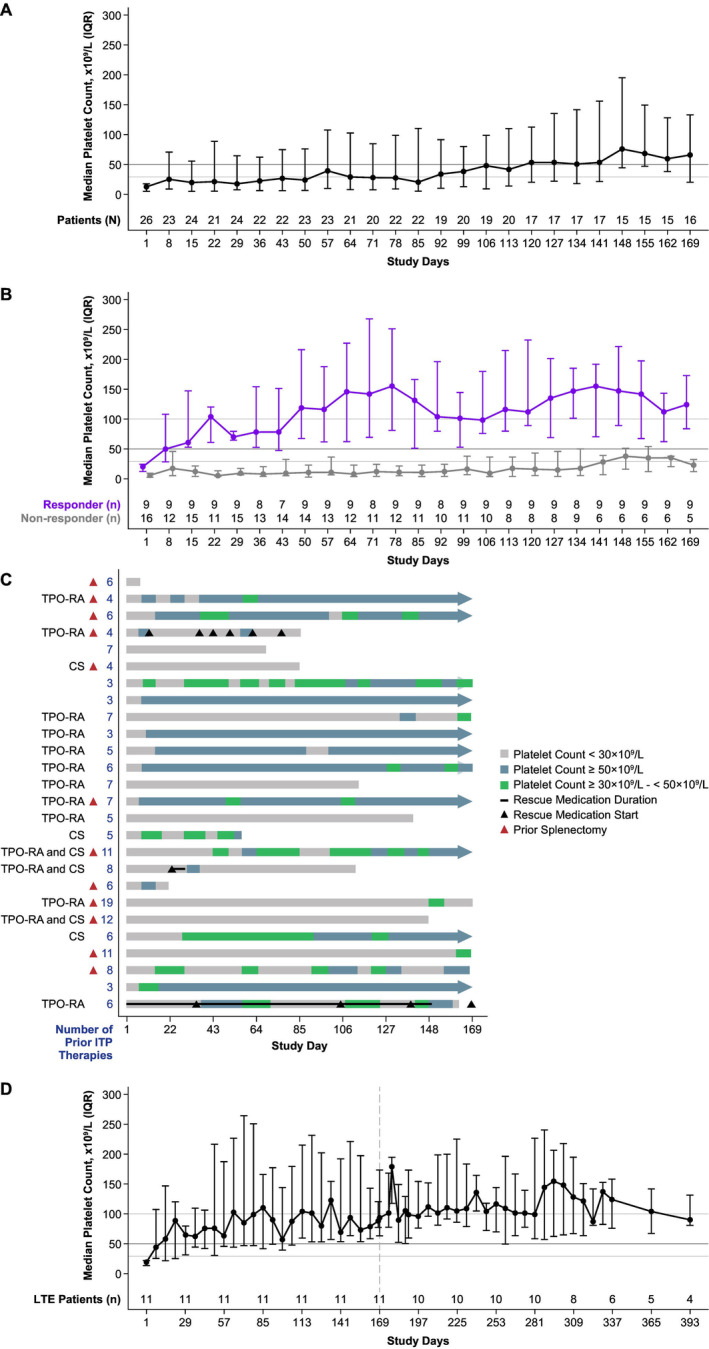
Efficacy outcomes all patients including median platelet counts (interquartile range [IQR]) from trial entry for all patients in the main treatment period (A) and by response (responder^a^ *n* = 9 and non‐responder *n* = 17) in the main treatment period (B), swimmer's plot^b^ for duration of response during the main treatment period (C), and median platelet counts (IQR) for long‐term extension (LTE) patients (*n* = 11) during the main and LTE treatment periods (D). ^a^Responder was defined as patients who met the primary endpoint. ^b^The swimmer's plot by patient per lane shows the duration of treatment with rilzabrutinib with or without concomitant corticosteroids (CS) and/or thrombopoietin receptor agonist (TPO‐RA) given at baseline, and platelet counts of < 30 × 10^9^/L (gray shading), ≥ 50 × 10^9^/L (blue shading) and ≥ 30 × 10^9^/L–< 50 × 10^9^/L (green shading). The red triangle represents prior splenectomy next to the number of prior ITP therapies (blue numbers) reported on the left side of each lane. The black triangle represents initiation of rescue medication and its duration with a black line. The blue arrow on the right of each lane signifies ongoing treatment after the 24‐week main treatment period.

The primary endpoint of durable platelet count response (≥ 50 × 10^9^/L on ≥ 8 of the last 12 weeks of treatment) was achieved in nine patients (35%; 95% CI, 17%–56%; Table 2). Median platelet counts generally increased over time, reaching 50 × 10^9^/L by study day 127 and were maintained above this level thereafter (Figure 1A). Ten (38%) patients had platelet counts of ≥ 50 × 10^9^/L by the day 15 visit (i.e., early responders). For nine (35%) patients achieving the primary endpoint, median platelet counts reached > 50 × 10^9^/L by day 8 and remained above that level through the last assessment, whereas levels for 17 (65%) non‐responders were below 30 × 10^9^/L for all assessments until day 148 (Figure 1B). A swimmer's plot profiles each patient, their baseline characteristics, use of concomitant medication at baseline, and platelet counts through the main treatment period (Figure 1C).

**TABLE 2:** Efficacy of rilzabrutinib 400 mg bid in patients with ITP.

	Patients (*N* = 26)
Primary endpoint	
Proportion of patients able to achieve platelet counts ≥ 50 × 10^9^/L on at least 8 of the last 12 weeks of the 24‐week treatment period without the use of rescue medication after 10 weeks of active treatment, *n* (% [95% CI])	9 (35% [17%, 56%])
Secondary endpoints	
Number of weeks with platelet count ≥ 50 × 10^9^/L or ≥ 30 × 10^9^/L and doubling from baseline in absence of rescue therapy over the 24‐week treatment period	
Mean (SD)	9.3 (10.1)
Median (IQR [range])	4.0 (0–21 [0–24])
Proportion of treated patients able to achieve two or more consecutive platelet counts separated by at least 5 days of ≥ 50 × 10^9^/L and an increase of platelet count of ≥ 20 × 10^9^/L from baseline without use of rescue medication in the 4 weeks prior to the latest elevated platelet count	11 (42%)
Number of weeks with platelet count ≥ 30 × 10^9^/L and doubling from baseline over the 24‐week treatment period	
Mean (SD) Median (IQR [range])	9.3 (10.1) 4.0 (0–21 [0–24])
Proportion of patients receiving rescue medication (% [95% CI])	3 (12% [2%, 30%])
Change from baseline at week 25 in IBLS	
Mean (SD)	−0.07 (0.12)
Median (IQR [range])	0 (−0.1 to 0 [−0.4 to 0])
Exploratory endpoints	
Proportion of patients who completed 24 weeks of treatment and demonstrated a platelet response defined as platelet counts ≥ 50 × 10^9^/L at 4 of the last 8 weeks of the active treatment period	11 (42%)
Proportion of patients (while on concomitant TPO‐RA) who have platelet counts, n/n (%)	
> 250 × 10^9^/L	2/14 (14%)
> 450 × 10^9^/L	1/14 (7%)
Time to first platelet count of ≥ 50 × 10^9^/L for all patients, days	
Median (IQR [95% CI])	95.5 (10–177 [15 to NA])
Time to first platelet count of ≥ 50 × 10^9^/L for patients who achieved platelet count ≥ 50 × 10^9^/L, days (*n* = 16)	
Median (IQR [range])	15 (8–75 [7–134])
Percentage of time with platelet counts ≥ 30 × 10^9^/L or ≥ 20 × 10^9^/L above baseline for all patients, days	
Mean (SD)	46 (44)
Median (IQR)	37 (0–92)
Percentage of time with platelet counts ≥ 30 × 10^9^/L or ≥ 20 × 10^9^/L above baseline for patients who achieved these platelet counts, days (*n* = 18)	
Mean (SD)	66 (37)
Median (IQR)	83 (33–100)

*Note*: Data are n (%), except where indicated.

Abbreviations: bid, twice daily; CI, confidence interval; IBLS, immune thrombocytopenia Bleeding Scale; IQR, interquartile range; ITP, immune thrombocytopenia; NA, not applicable (i.e., not reached); SD, standard deviation; TPO‐RA, thrombopoietin receptor agonist.

Among patients with platelet counts ≥ 50 × 10^9^/L at any time in the first 2 weeks, ≥ 50 × 10^9^/L at any time in the first 8 weeks, or platelet counts ≥ 30 × 10^9^/L and ≥ 20 × 10^9^/L above baseline over the first 2 weeks, the proportion of patients who met the primary endpoint was high at 80%, 67%, or 62%, respectively. Conversely, patients not meeting these early threshold levels were more likely to be non‐responders with 94%, 93%, or 92% of patients, respectively, not meeting the primary endpoint (Table S1).

Subgroup analysis of the primary endpoint by baseline factors, prior treatment, and concomitant therapy showed consistent results for all patients and subgroups with > 10 participants (Figure S2). Notably, patients with baseline platelet count ≥ 15 × 10^9^/L had a higher primary endpoint response (58% for 7/12 patients) than patients with baseline platelet count < 15 × 10^9^/L (14% for 2/14 patients). Median platelet counts over time were similar for patients receiving rilzabrutinib monotherapy or with concomitant ITP therapy (Figure S3).

Platelet counts ≥ 50 × 10^9^/L or ≥ 30 × 10^9^/L and doubling from baseline in the absence of rescue therapy were sustained for a mean of 9.3 weeks (standard deviation [SD], 10.1; Table 2). Two or more consecutive platelet counts ≥ 50 × 10^9^/L and increased ≥ 20 × 10^9^/L from baseline in the 4 weeks prior to the latest elevated platelet count was achieved in 11 (42%) patients.

Of 26 enrolled patients, 11 (42%) patients completed 24 weeks of treatment and were eligible to enter the LTE (including nine patients meeting the primary endpoint). Median platelet counts for LTE patients were maintained above 80 × 10^9^/L during the LTE period (Figure 1D).

Three (12%) patients received rescue medication during the 24‐week main treatment period to manage deterioration in platelet counts that in the investigator's opinion put the patient at risk for a serious adverse event (SAE). Concomitant rescue medication with intravenous immunoglobulins (not otherwise specified) was given to two patients, and prednisolone and eltrombopag (prohibited medication; the patient was not a responder) to one patient each, without interruption of rilzabrutinib. No patient in the LTE received rescue medication.

At baseline, all patients had a mean IBLS score of 0.27 (SD, 0.27) that decreased to 0.06 (SD, 0.08) at week 25, with a mean change from baseline at week 25 of −0.07 (SD, 0.12), indicating decreased bleeding. At baseline, the bleeding score for skin was two in seven patients per medical history and six per physical examination; at week 25, no patients had a score of two for skin (Table S2).

Additional exploratory efficacy endpoints are detailed in Table 2. In 14 patients receiving concomitant TPO‐RA, two patients had platelet counts that exceeded 250 × 10^9^/L and one patient had counts that exceeded 450 × 10^9^/L. For 16 patients who had a platelet count ≥ 50 × 10^9^/L, median time to first platelet count ≥ 50 × 10^9^/L was 15 days (IQR, 8–75; Table 2; Figure S4).

### 3.4 Safety

In the main treatment period, median duration of treatment was 167 days (IQR, 112–168), with a median compliance rate of 99% (IQR, 96%–100%). Twenty‐two (85%) patients experienced an AE due to any cause and there were no deaths (Table 3). Three patients reported SAEs of grade 4 thrombocytopenia, grade 3 subcutaneous abscess due to an underlying medical condition, and concurrent grade 3 post‐procedural hemorrhage and grade 3 syncope (appendix); none were considered related to treatment by site investigators, and all resolved. Sixteen (62%) patients had at least one treatment‐related AE, the most common were nine (35%) patients with diarrhea, six (23%) with headache, and four (15%) with nausea. Most related AEs were grade 1. One patient had a treatment‐related grade 3 serum creatinine phosphokinase increase on day 29 that lasted for 7 days with no change in rilzabrutinib dose. There was no treatment‐related grade ≥ 2 bleeding/thrombotic events or infections, SAEs, or deaths.

**TABLE 3:** Adverse events by maximum grade during the main treatment period (*N* = 26).

	Adverse events due to any cause				Treatment‐related adverse events			
	Any grade	Grade 1	Grade 2	Grade ≥ 3^a^	Any grade	Grade 1	Grade 2	Grade 3^b^
Any adverse event	22 (85%)	21 (81%)	13 (50%)	4 (15%)	16 (62%)	14 (54%)	4 (15%)	1 (4%)
Diarrhea	12 (46%)	9 (35%)	3 (12%)	0	9 (35%)	7 (27%)	2 (8%)	0
Headache	9 (35%)	8 (31%)	1 (4%)	0	6 (23%)	5 (19%)	1 (4%)	0
Nausea	6 (23%)	6 (23%)	0	0	4 (15%)	4 (15%)	0	0
Upper abdominal pain	5 (19%)	3 (12%)	2 (8%)	0	1 (4%)	0	1 (4%)	0
Dyspepsia	5 (19%)	4 (15%)	1 (4%)	0	2 (8%)	1 (4%)	1 (4%)	0
Gastroesophageal reflux disease	4 (15%)	2 (8%)	2 (8%)	0	2 (8%)	2 (8%)	0	0
Fatigue	3 (12%)	2 (8%)	1 (4%)	0	0	0	0	0
Abdominal discomfort	2 (8%)	2 (8%)	0	0	1 (4%)	1 (4%)	0	0
Anemia	2 (8)	1 (4)	0	1 (4)	0	0	0	0
Serum creatinine phosphokinase increased	1 (4%)	0	0	1 (4%)	1 (4%)	0	0	1 (4%)
Syncope	1 (4%)	0	0	1 (4%)	0	0	0	0
Thrombocytopenia	1 (4%)	0	0	1 (4%)^a^	0	0	0	0
Dyspnea	1 (4%)	0	1 (4%)	0	1 (4%)	0	1 (4%)	0
Head discomfort	1 (4%)	0	1 (4%)	0	1 (4%)	0	1 (4%)	0
Vomiting	1 (4%)	0	1 (4%)	0	1 (4%)	0	1 (4%)	0
Abnormal skin odor	1 (4%)	1 (4%)	0	0	1 (4%)	1 (4%)	0	0
Abnormal weight loss	1 (4%)	1 (4%)	0	0	1 (4%)	1 (4%)	0	0
Inappropriate schedule of product administration	1 (4%)	1 (4%)	0	0	1 (4%)	1 (4%)	0	0
Muscle spasms	1 (4%)	1 (4%)	0	0	1 (4%)	1 (4%)	0	0
Pain	1 (4%)	1 (4%)	0	0	1 (4%)	1 (4%)	0	0
Bleeding events								
Contusion	5 (19%)	3 (12%)	2 (8%)	0	0	0	0	0
Epistaxis	2 (8%)	1 (4%)	1 (4%)	0	0	0	0	0
Post‐procedural hemorrhage	1 (4%)	0	0	1 (4%)	0	0	0	0
Blood urine present	1 (4%)	0	1 (4%)	0	0	0	0	0
Gingival bleeding	1 (4%)	1 (4%)	0	0	0	0	0	0
Infections and infestations								
COVID‐19^c^	5 (19%)	3 (12%)	2 (8%)	0	0	0	0	0
Upper respiratory tract infection	4 (15%)	2 (8%)	2 (8%)	0	0	0	0	0
Subcutaneous abscess	1 (4%)	0	0	1 (4%)	0	0	0	0
Gastroenteritis	1 (4%)	0	1 (4%)	0	0	0	0	0
Meningitis aseptic	1 (4%)	0	1 (4%)	0	0	0	0	0
Urinary tract infection	1 (4%)	0	1 (4%)	0	0	0	0	0
Sinusitis	1 (4%)	1 (4%)	0	0	0	0	0	0
Viral upper respiratory tract infection	1 (4%)	1 (4%)	0	0	0	0	0	0

*Note*: Adverse event data are shown by system organ class and preferred term for the number of patients (% of patients). Table includes all treatment‐related adverse events, any‐cause grade 1–2 adverse events occurring in at least 10% of patients, all bleeding‐ and infection‐related events, and all grade 3 or 4 events (there were 0 grade 5 events). Patients may have had more than one adverse event.

^a^ Included one patient with grade 4 thrombocytopenia.

^b^ One patient had a treatment‐related grade 3 adverse event; no patients had grade 4 related events.

^c^ Five patients had COVID‐19 infections (one of whom had a prior splenectomy); none were related to treatment, all patients recovered without treatment interruption or modification.

As bleeding is a known safety risk in ITP due to thrombocytopenia,^1^ these events were evaluated separately (Table 3). Four (15%) patients had grade ≥ 2 bleeding events, including grade 2 contusion, epistaxis, and blood urine present, and grade 3 post‐procedural hemorrhage, which was an SAE leading to dose interruption on day 5. All events were resolved and none were considered related to treatment by the site investigator.

Thirteen (50%) patients reported infection‐related AEs during the main treatment period; all were grade 1 or 2, except one grade 3 SAE of subcutaneous abscess (Table 3). This patient had an underlying subcutaneous abscess that worsened, and the site investigator decided to stop rilzabrutinib permanently on day 84 due to the grade 3 subcutaneous abscess. None of these events were considered related to treatment by the site investigator and all resolved.

For 11 LTE patients, median duration of LTE treatment was 182 days (IQR, 125–323), with a median compliance rate of 98% (IQR, 96–100). Eight of 11 (73%) LTE patients experienced an AE due to any cause in the LTE period (Table S3). The only AEs due to any cause occurring in > 1 patient were epistaxis (one grade 1, one grade 2) and nausea (two grade 1); all others were observed in one patient only (Table S3). One patient had grade 3 cholelithiasis and grade 2/3 serum gamma‐glutamyl transferase increase unrelated to treatment per the site investigator assessment. One patient with a medical history of transient ischemic attack, myocardial infarction, diabetes mellitus type 2, hypertension, and hypercholesterolemia experienced a grade 2 transient ischemic attack designated an SAE, however, was not related to study treatment per the site investigator assessment. Only one (9%) patient had a treatment‐related event of grade 1 diarrhea that resolved with no change in rilzabrutinib dose in the LTE. There were no treatment‐related grade ≥ 2 bleeding/thrombotic or infectious events, SAEs, or deaths, and no LTE patients discontinued rilzabrutinib.

### 3.5 Exploratory Endpoints

Exploratory evaluation of hemolysis marker haptoglobin and immunoglobulins were within normal ranges at baseline and week 25 following rilzabrutinib treatment (Table S4). Median changes from baseline to week 25 showed an increase in haptoglobin levels from 0.75 to 1.11 g/L (median change 0.33 g/L), decrease in immunoglobulin G (IgG) from 10.9 to 9.2 g/L (median change −1.55 g/L), and minimal to no changes in IgG1, IgG4, IgM, or IgE levels. TPO was above normal at baseline (median 175 ng/L [IQR, 117–243]), decreased at week 25 (median 110 ng/L [IQR, 58–176]), showing a median change from baseline to week 25 of −59 ng/L (IQR, −118 to 11; Table S4).

Clinically meaningful improvements in ITP‐PAQ HRQOL scores (i.e., minimum important difference [MID]) [20] for the median change from baseline to week 25 per were observed in domains for women's reproductive health (median MID change, 21), overall HRQOL (13), fatigue/sleep (13), and activity (13) scales (Table S5, Figure S5). Numerical improvements in overall HRQOL for the change from baseline to week 25 were also observed using EQ‐VAS and with ITP‐PAQ for psychological, symptoms, social activity, and bother‐physical health domains.

## 4 Discussion

Rilzabrutinib treatment led to rapid and durable platelet count responses in patients with ITP who were unresponsive to a median of six (up to 19) prior ITP therapies. Durable platelet count responses (primary endpoint) were observed in nine patients; these nine patients plus two additional patients met the eligibility criteria to continue receiving rilzabrutinib in the LTE. Platelet counts reached a median of 50 × 10^9^/L in nine responding patients by day 8 of rilzabrutinib treatment and were sustained above a median of 80 × 10^9^/L in all 11 LTE patients throughout the LTE period.

In a post hoc subgroup analysis of platelet response, achievement of the primary endpoint was consistent across most subgroups of baseline factors and response to prior therapy, except lower responses for patients with baseline platelet counts below 15 × 10^9^/L. A major challenge in ITP is maintenance of an effective therapeutic response over time. As observed here in the subgroup analysis, despite all patients receiving prior corticosteroids and 12 (46%) patients having a prior splenectomy, a consistent response was observed over time to rilzabrutinib, whether given as monotherapy or in combination with other ITP medication. This is clinically important as these patients entered the trial with a history of inadequate responses to multiple prior (or concomitant) lines of ITP therapy.

Relative to ITP patients in the part A study of dose‐escalated rilzabrutinib previously reported by Kuter and colleagues [13], part B patients were older (median age 57 vs. 50 years for part A), had a longer duration of ITP (median 10.3 vs. 6.3 years), received more prior ITP therapies (median 6 vs. 4), and more patients were splenectomized (46% vs. 25%). Despite the potentially harder to treat patient characteristics in part B, both studies showed comparable overall platelet response (~40%) and rapid time to response (~2 weeks). Interestingly, despite the relatively higher prior treatment refractoriness of part B compared to part A patients, more part B patients achieved the LTE eligibility criteria (11/26 [42%] vs. 16/60 [27%] part A).

The safety profile of rilzabrutinib reported predominantly grade 1 treatment‐related AEs (27% diarrhea, 19% headache, 15% nausea) that resolved, allowing for continued treatment. All other related AEs were present in ≤ 2 (< 10%) patients. The only related grade 3 AE was an increase in serum creatinine phosphokinase levels, which resolved without interruption of rilzabrutinib. A common safety concern for ITP patients is an elevated risk for bleeding due to thrombocytopenia [1]. However, there were no bleeding, thrombotic, or infectious events related to rilzabrutinib. Decreased bleeding scale scores at week 25 suggest improvement in the risk for bleeding with rilzabrutinib. There was also no evidence of safety signals typically associated with irreversible BTK inhibitor class effects, eg, bleeding, neutropenia, anemia, thrombotic events, infection, or atrial fibrillation [21, 22].

Current second‐line treatment recommendations in ITP include the use of TPO‐RA, splenectomy, or immunomodulators (e.g., rituximab, fostamatinib) [5, 23]. Although these options have shown robust evidence for treating ITP, their long‐term effectiveness is limited by incomplete mechanistic activity for specifically targeting disease pathophysiology, increased risks for bleeding/thromboembolic or surgery‐related events, elevated toxicity due to continuous use, and/or impaired HRQOL [24]. Besides immune‐mediated destruction and impaired platelet production, patients with active ITP (vs remission or non‐ITP controls) show evidence of significantly higher expression of NLRP3 inflammasome, adaptor protein ASC, and plasma IL‐18 levels, and increased caspase‐1 activity and markers of endothelial cell activation and neutrophil extracellular trap formation [25, 26]. Independent of platelet count, elevated ITP‐associated inflammatory states may contribute to increased intrinsic hypercoagulability and downstream HRQOL effects (e.g., fatigue) [25, 27]. While a direct link has yet to be shown with rilzabrutinib in patients with ITP, other preclinical studies have identified a role for BTK as a direct regulator of the NLRP3 inflammasome in innate immunity [28], suggesting the potential for therapeutic effects beyond increasing platelet counts in ITP that may include ITP‐associated complications attributable to chronic inflammation, such as fatigue and thromboembolism.

Rilzabrutinib provides the convenience of oral administration coupled with multiple mechanisms of action applicable to ITP including inhibiting B‐cell activation, interrupting antibody‐coated phagocytosis by Fcγ receptor signaling, and potentially inducing sustained anti‐inflammatory effects [10]. Phase 2 results to date with rilzabrutinib show a robust efficacy signal and acceptable safety profile in patients with difficult to treat ITP by preventing severe bleeding episodes, maintaining target platelet levels (> 30 × 10^9^/L) to minimize bleeding risk, and providing acceptable safety during both the main and LTE treatment periods [5]. Continued follow‐up will further evaluate the durability of response and tolerability with rilzabrutinib. Additionally, rilzabrutinib showed clinically meaningful HRQOL improvements at week 25 in multiple domains per MID values [20].

Although the current part B study is limited by the single‐arm nature of the trial design with a small number of adult patients, the observed rapid and sustained therapeutic effects in previously treated ITP patients suggest clinically meaningful efficacy. The safety and efficacy profile of rilzabrutinib at the selected dose of 400 mg bid observed in part B was consistent with part A [13, 14]. Results from the ongoing phase 3, placebo‐controlled LUNA 3 study will further assess the magnitude and durability of response, as well as safety profile, with rilzabrutinib in previously treated adult and adolescent patients with primary ITP [29, 30].

## Author Contributions

N.C., M.Y., and D.J.K. contributed to the conception and design of the trial. N.C., A.J.G.J., R.B., J.M., M.S., M.D.T., M.G., P.F.Y., V.M., C.P., M.K., I.G., L.H.B., T.B.G., and D.J.K. contributed to the data acquisition and collection, and enrolling and treating patients. M.Y., A.D., and R.M.D. developed the study's statistical analysis plan. A.D., M.Y., D.J.K., and R.M.D. analyzed and interpreted the data. C.P., M.K., M.Y., A.D., and D.J.K. wrote the manuscript. All authors reviewed and approved the manuscript. All authors had access to, verified, and interpreted the primary clinical data; critically reviewed and revised the manuscript, and approved the final version for submission. All authors attest that the trial was done in accordance with the protocol and vouch for the accuracy and completeness of the data and analyses. All authors had final responsibility for the decision to submit for publication.

## Ethics Statement

The trial was conducted in accordance with the principles of the Declaration of Helsinki and International Council for Harmonization Good Clinical Practice E6 requirements. The protocol and informed‐consent documents were approved by the Institutional Review Board and International Ethics Committees at each participating institution.

## Consent

All patients provided written informed consent.

## Conflicts of Interest

Nichola Cooper: reports support for present manuscript from Sanofi; partially supported from the imperial NIHR BRC; research support/grant funding for institution for separate studies from Novartis (ITP) and Rigel (COVID‐19); consulting fees for treatments for ITP from argenyx, Novartis, Sanofi, and Sobi; honoraria for lectures at educational meetings from Novartis, Sanofi, and Sobi; and support for travel and hotel accommodations for ASH 2023 and EHA 2024 from Novartis. A. J. Gerard Jansen: reports grant for ultrasound in hemophilia care from Sobi; Heimburger Award from CSL Behring; consulting fees from Amgen and Novartis; payment or honoraria for lectures, presentations, speakers bureau, manuscript writing or educational events from Amgen, Novartis, and Sobi; support for attending meetings and/or travel from 3SBio, Amgen, Novartis, and Sobi; patent P133951EP00 granted 01‐11‐2022; participation on a data safety monitoring board or advisory board from Novartis; unpaid leadership or fiduciary role in other board, society, committee, or advocacy group as chair NVvH ITP working group, secretary NVvH scientific committee NVB, and Erasmus MC ethics committee; and receipt of drugs for clinical trials from argenx, Principia, and Sanofi. Robert Bird: no financial disclosures to report. Jiří Mayer: support for the present manuscript and grants or contracts from Sanofi. Michelle Sholzberg: reports honoraria from Medison and Sobi. Michael D. Tarantino: consulting fees from Amgen, Biomarin, Dova Pharmaceuticals, Genentech, Novo Nordisk, Octapharma, Pfizer, Sanofi, Sobi‐Swedish Orphan Biovirtum, Takeda, and USB Biosciences; payment or honoraria from Amgen, Biomarin, Genentech, Novartis, Sanofi, and Sobi‐Swedish Orphan Biovirtum; and participation on data safety monitoring board or advisory board for Octapharma and Takeda. Mamta Garg: payment or honoraria for lectures, presentations, speakers bureaus, manuscript writing or educational events from Amgen, AOP Orphan Pharmaceuticals, Janssen J&J, Novartis, and Pfizer; support for attending meetings and/or travel from Amgen, AOP Orphan Pharmaceuticals, Bristol Myers Squibb (Celgene), Janssen J&J, Novartis, Sanofi, and Stemline Therapeutics; and participation on data safety monitoring board or advisory board for Amgen, Bristol Myers Squibb (Celgene), CTI BioPharma, Janssen J&J, and Stemline Therapeutics. Paula F. Ypma: no financial disclosures to report. Vickie McDonald: payment or honoraria for lectures, presentations, speakers bureaus, manuscript writing or educational events from Amgen, Grifols, Novartis, and Sobi; and support for attending meetings and/or travel from Novartis. Charles Percy: no financial disclosures to report. Milan Košťál: no financial disclosures to report. Isaac Goncalves: no financial disclosures to report. Lachezar H. Bogdanov: no financial disclosures to report. Terry B. Gernsheimer: research support for patient enrollment in LUNA phase 2 study; consulting fees from Amgen, Alpine Immune Sciences, argenx, Bioproducts Laboratory (Kedrion), Cellphire, and Sanofi; payment or honoraria for presentation/video from Sanofi; support for travel to advisory board meeting from Novartis (June 2024) and Sanofi (July 2022); and participation on advisory board for Sanofi (July 2022). Remco Diab, Mengjie Yao, and Ahmed Daak report current employment and current equity holders in the publicly held company Sanofi. David J. Kuter: reports research support to institution from Sanofi; consulting from Sanofi; grants or contracts to the institution from BioCryst, HUTCHMED, Novartis, Principia, and Sanofi; royalties from UpToDate; consulting fees from, payment or honoraria for lectures, presentations, speakers bureaus, manuscript writing or education events, and receipt of equipment, materials, drugs, medical writing, gifts or other services from Alexion, Alnylam, Alpine, Amgen, Apellis, argenx, BioCryst, Bristol Myers Squibb, Caremark, Cellularity, Cellphire, Chugai, Jiangsu Hengrui, HUTCHMED, Immunovant, Inmagene Bio, Medscape, Merck Sharp & Dohme, Novartis, PER, Pfizer, Platelet Disorder Support Association (PDSA), Principia, Regeneron, Rigel, Sanofi, Seismic, Sobi, Takeda, UpToDate, Verve; and other non‐financial interests with the PDSA medical advisory board.

## Supporting information


Data S1.

## Data Availability

Qualified researchers may request access to patient‐level data and related study documents including the clinical study report, study protocol with any amendments, blank case report form, statistical analysis plan, and dataset specifications. Patient‐level data will be anonymized and study documents will be redacted to protect the privacy of our trial participants. Further details on Sanofi's data sharing criteria, eligible studies, and process for requesting access can be found at: https://www.vivli.org/.

## References

[1] K. Adelborg , N. R. Kristensen , M. Norgaard , et al., “Cardiovascular and Bleeding Outcomes in a Population‐Based Cohort of Patients With Chronic Immune Thrombocytopenia,” Journal of Thrombosis and Haemostasis 17, no. 6 (2019): 912–924. 30933417 10.1111/jth.14446

[2] N. Cooper and W. Ghanima , “Immune Thrombocytopenia,” New England Journal of Medicine 381, no. 10 (2019): 945–955. 31483965 10.1056/NEJMcp1810479

[3] F. Efficace , F. Mandelli , P. Fazi , et al., “Health‐Related Quality of Life and Burden of Fatigue in Patients With Primary Immune Thrombocytopenia by Phase of Disease,” American Journal of Hematology 91, no. 10 (2016): 995–1001. 27351715 10.1002/ajh.24463

[4] F. Rodeghiero , R. Stasi , T. Gernsheimer , et al., “Standardization of Terminology, Definitions and Outcome Criteria in Immune Thrombocytopenic Purpura of Adults and Children: Report From an International Working Group,” Blood 113, no. 11 (2009): 2386–2393. 19005182 10.1182/blood-2008-07-162503

[5] D. Provan , D. M. Arnold , J. B. Bussel , et al., “Updated International Consensus Report on the Investigation and Management of Primary Immune Thrombocytopenia,” Blood Advances 3, no. 22 (2019): 3780–3817. 31770441 10.1182/bloodadvances.2019000812 PMC6880896

[6] G. Lopez‐Herrera , A. Vargas‐Hernandez , M. E. Gonzalez‐Serrano , et al., “Bruton's Tyrosine Kinase—An Integral Protein of B Cell Development That Also Has an Essential Role in the Innate Immune System,” Journal of Leukocyte Biology 95, no. 2 (2014): 243–250. 24249742 10.1189/jlb.0513307

[7] L. J. Crofford , L. E. Nyhoff , J. H. Sheehan , and P. L. Kendall , “The Role of Bruton's Tyrosine Kinase in Autoimmunity and Implications for Therapy,” Expert Review of Clinical Immunology 12, no. 7 (2016): 763–773. 26864273 10.1586/1744666X.2016.1152888 PMC5070917

[8] M. Montillo , S. O'Brien , A. Tedeschi , et al., “Ibrutinib in Previously Treated Chronic Lymphocytic Leukemia Patients With Autoimmune Cytopenias in the RESONATE Study,” Blood Cancer Journal 7, no. 2 (2017): e524. 28157216 10.1038/bcj.2017.5 PMC5386339

[9] K. A. Rogers , A. S. Ruppert , A. Bingman , et al., “Incidence and Description of Autoimmune Cytopenias During Treatment With Ibrutinib for Chronic Lymphocytic Leukemia,” Leukemia 30, no. 2 (2016): 346–350. 26442611 10.1038/leu.2015.273 PMC4986508

[10] C. L. Langrish , J. M. Bradshaw , M. R. Francesco , et al., “Preclinical Efficacy and Anti‐Inflammatory Mechanisms of Action of the Bruton Tyrosine Kinase Inhibitor Rilzabrutinib for Immune‐Mediated Disease,” Journal of Immunology 206, no. 7 (2021): 1454–1468. 10.4049/jimmunol.2001130 PMC7980532 33674445

[11] T. D. Owens , K. A. Brameld , E. J. Verner , et al., “Discovery of Reversible Covalent Bruton's Tyrosine Kinase Inhibitors PRN473 and PRN1008 (Rilzabrutinib),” Journal of Medicinal Chemistry 65, no. 7 (2022): 5300–5316. 35302767 10.1021/acs.jmedchem.1c01170

[12] P. F. Smith , J. Krishnarajah , P. A. Nunn , et al., “A Phase I Trial of PRN1008, a Novel Reversible Covalent Inhibitor of Bruton's Tyrosine Kinase, in Healthy Volunteers,” British Journal of Clinical Pharmacology 83, no. 11 (2017): 2367–2376. 28636208 10.1111/bcp.13351 PMC5651318

[13] D. J. Kuter , M. Efraim , J. Mayer , et al., “Rilzabrutinib, an Oral BTK Inhibitor, in Immune Thrombocytopenia,” New England Journal of Medicine 386, no. 15 (2022): 1421–1431. 35417637 10.1056/NEJMoa2110297

[14] D. J. Kuter , J. Mayer , M. Efraim , et al., “Long‐Term Treatment With Rilzabrutinib in Patients With Immune Thrombocytopenia,” Blood Advances 8, no. 7 (2024): 1715–1724. 38386978 10.1182/bloodadvances.2023012044 PMC10997915

[15] F. Rodeghiero , M. Michel , T. Gernsheimer , et al., “Standardization of Bleeding Assessment in Immune Thrombocytopenia: Report From the International Working Group,” Blood 121, no. 14 (2013): 2596–2606. 23361904 10.1182/blood-2012-07-442392

[16] L. K. Page , B. Psaila , D. Provan , et al., “The Immune Thrombocytopenic Purpura (ITP) Bleeding Score: Assessment of Bleeding in Patients With ITP,” British Journal of Haematology 138, no. 2 (2007): 245–248. 17542983 10.1111/j.1365-2141.2007.06635.x

[17] N. Devlin , D. Parkin , and B. Janssen , “An Introduction to EQ‐5D Instruments and Their Applications,” in Methods for Analysing and Reporting EQ‐5D Data (Cham (CH): Springer, July 2020). 33347096

[18] Y. S. Feng , T. Kohlmann , M. F. Janssen , and I. Buchholz , “Psychometric Properties of the EQ‐5D‐5L: A Systematic Review of the Literature,” Quality of Life Research 30, no. 3 (2021): 647–673. 33284428 10.1007/s11136-020-02688-y PMC7952346

[19] S. D. Mathias , J. B. Bussel , J. N. George , R. McMillan , G. J. Okano , and J. L. Nichol , “A Disease‐Specific Measure of Health‐Related Quality of Life for Use in Adults With Immune Thrombocytopenic Purpura: Its Development and Validation,” Health and Quality of Life Outcomes 5 (2007): 11. 17316442 10.1186/1477-7525-5-11 PMC1808052

[20] S. D. Mathias , S. K. Gao , M. Rutstein , C. F. Snyder , A. W. Wu , and D. Cella , “Evaluating Clinically Meaningful Change on the ITP‐PAQ: Preliminary Estimates of Minimal Important Differences,” Current Medical Research and Opinion 25, no. 2 (2009): 375–383. 19192982 10.1185/03007990802634119

[21] C. Aguilar , “Ibrutinib‐Related Bleeding: Pathogenesis, Clinical Implications and Management,” Blood Coagulation & Fibrinolysis 29, no. 6 (2018): 481–487. 29995658 10.1097/MBC.0000000000000749

[22] P. von Hundelshausen and W. Siess , “Bleeding by Bruton Tyrosine Kinase‐Inhibitors: Dependency on Drug Type and Disease,” Cancers (Basel) 13, no. 5 (2021): 1103. 33806595 10.3390/cancers13051103 PMC7961939

[23] C. Neunert , D. R. Terrell , D. M. Arnold , et al., “American Society of Hematology 2019 Guidelines for Immune Thrombocytopenia,” Blood Advances 3, no. 23 (2019): 3829–3866. 31794604 10.1182/bloodadvances.2019000966 PMC6963252

[24] D. J. Kuter , “Novel Therapies for Immune Thrombocytopenia,” British Journal of Haematology 196, no. 6 (2022): 1311–1328. 34611885 10.1111/bjh.17872

[25] L. Garabet , C. E. Henriksson , M. L. Lozano , et al., “Markers of Endothelial Cell Activation and Neutrophil Extracellular Traps Are Elevated in Immune Thrombocytopenia but Are Not Enhanced by Thrombopoietin Receptor Agonists,” Thrombosis Research 185 (2020): 119–124. 31805421 10.1016/j.thromres.2019.11.031

[26] J. Qiao , Y. Liu , X. Li , et al., “Elevated Expression of NLRP3 in Patients With Immune Thrombocytopenia,” Immunologic Research 64, no. 2 (2016): 431–437. 26306997 10.1007/s12026-015-8686-5

[27] Q. A. Hill and A. C. Newland , “Fatigue in Immune Thrombocytopenia,” British Journal of Haematology 170, no. 2 (2015): 141–149. 25823426 10.1111/bjh.13385

[28] A. N. R. Weber , Z. Bittner , X. Liu , T. M. Dang , M. P. Radsak , and C. Brunner , “Bruton's Tyrosine Kinase: An Emerging Key Player in Innate Immunity,” Frontiers in Immunology 8 (2017): 1454. 29167667 10.3389/fimmu.2017.01454 PMC5682317

[29] National Institutes of Health , “Study to Evaluate Rilzabrutinib in Adults and Adolescents With Persistent or Chronic Immune Thrombocytopenia (ITP) (LUNA 3) (NCT04562766),” accessed August 14, 2023, https://clinicaltrials.gov/ct2/show/NCT04562766.

[30] EU Clinical Trials Register , “A Phase 3, Multicenter, Randomized, Double‐Blind, Placebo‐Controlled, Parallel‐Group Study With an Open‐Label Extension to Evaluate the Efficacy and Safety of Oral Rilzabrutinib (PRN1008) in Adults and Adolescents With Persistent or Chronic Immune Thrombocytopenia (ITP) (EudraCT Number: 2020‐002063‐60),” accessed August 14, 2023, https://www.clinicaltrialsregister.eu/ctr‐search/search?query=2020‐002063‐60.

