# Supplementary material for: Efficacy and Safety Results With Rilzabrutinib, an Oral Bruton Tyrosine Kinase Inhibitor, in Patients With Immune Thrombocytopenia: Phase 2 Part B Study
Source: Am J Hematol. 2025 Jan 22;100(3):439–49. doi: 10.1002/ajh.27539 (PMC11803537; doi:10.1002/ajh.27539)
Supplement: Supplementary file 1 — Data S1. [file AJH-100-439-s001.docx]

**Supplemental Material: Appendix**

**Efficacy and safety results with rilzabrutinib, an oral Bruton tyrosine kinase inhibitor, in patients with immune thrombocytopenia: Phase 2 part B study**

**Cooper et al.**

**Table of Contents**

**Supplemental Methods**

**Supplemental Results**

**Supplemental Tables**

Table S1: Predictive platelet response following rilzabrutinib treatment during the 24-week main treatment period

Table S2: IBLS bleeding scale scores per location at the end of the main treatment period

Table S3: All adverse events by maximum grade during the long-term extension period (n=11)

Table S4: Effect of rilzabrutinib on markers of hemolysis (haptoglobin), immunoglobulin G (IgG), IgG1, IgG4, IgM, IgE, and thrombopoietin (TPO) levels during the main treatment period

Table S5: ITP-PAQ domain scores for the effect of rilzabrutinib on HRQOL

Table S6: LUNA 2 study investigators and site locations

**Supplemental Figures**

Figure S1: CONSORT diagram

Figure S2: Subgroup analysis of platelet response by baseline factors and concomitant therapy

Figure S3: Median (IQR) platelet counts over time with rilzabrutinib monotherapy or rilzabrutinib plus concomitant ITP therapy

Figure S4: Time to first platelet count ≥50×10^9^/L

Figure S5: Clinically significant and numerical changes in ITP-PAQ domain scores over time (A) and change from baseline to week 25 (B) for the effect of rilzabrutinib on HRQOL

**Supplemental References**

**Supplemental Methods**

**Eligibility criteria**

Adequate hematologic, hepatic, and renal function defined as absolute neutrophil count ≥1.5×10^9^/L, hemoglobin >9 g/dL, aspartate transferase/alanine transferase ≤1.5×upper limit of normal (ULN), albumin ≥3 g/dL, total bilirubin ≤1.5×ULN, and estimated glomerular filtration rate >50 mL/min (Cockcroft and Gault method) were required. Females of reproductive potential were required to use highly effective means of contraception during the study; if postmenopausal, then menopause was confirmed by follicular stimulating hormone testing.

Exclusion criteria consisted of pregnant/lactating females; electrocardiogram findings of QTcF >450 msec for males or >470 msec for females or poorly controlled atrial fibrillation; history of active malignancy within 5 years requiring chemotherapy or surgery (except non-melanoma skin cancer); transfusion, immunosuppressants (not including corticosteroids), or anticoagulants and platelet aggregation inhibiting drugs within 2 weeks; use of immune thrombocytopenia (ITP) rescue medication, investigational drug, or live vaccine within 4 weeks; use of rituximab or splenectomy within 3 months; ongoing need for proton pump inhibitor; use of concomitant strong to moderate cytochrome P450 3A inducer/inhibitor or sensitive substrate drugs within the longer of 3 days or 5 half-lives; history of solid organ transplant or recurring serious infection requiring antibiotic therapy; positive for human immunodeficiency, hepatitis B or C virus; myelodysplastic syndrome; or planned surgery during treatment.

**Procedures**

Patients could receive another ITP therapy (eg, intravenous immunoglobulin [IVIg], high-dose steroids, platelet transfusion, or anti-D immunoglobulin transfusion) for a significant safety event to prevent deterioration of their platelet count that in the investigator’s opinion put the patient at a significant safety risk. Rituximab or other anti-CD20 medications, vincristine, or other immunosuppressants, and initiation or increased in eltrombopag, romiplostim, or avatrombopag were not permitted as rescue therapy while receiving rilzabrutinib. Patients receiving rescue therapy could continue rilzabrutinib if no stopping rules applied and the investigator agreed to continue treatment. Individual stopping rules for rilzabrutinib treatment included pregnancy, abnormal liver function (grade ≥3 elevation of alanine aminotransferase [ALT] and/or aspartate aminotransferase [AST]; ALT or AST >3x upper limit of normal [ULN] with total bilirubin >2x ULN without ALT >2x ULN in the absence of another cause), and any situation that results in undue safety risk to the patient according to the investigator.

**Supplemental Results**

**Patient disposition**

Eleven (42%) patients discontinued during the 24-week treatment period due to lack of response (n=5); adverse events unrelated to treatment (n=3, appendix); and 1 patient each due to non-compliance, erroneous enrollment (hepatitis C antibody positive at screening), and combined lack of response and adverse event (treatment-related grade 1 diarrhea). For the three patients who discontinued treatment due to adverse events unrelated to treatment, the first patient had pre-existing grade 1 urinary tract infection, grade 3 hemorrhage, and grade 3 back pain; this patient received rilzabrutinib for 7 days. The second unrelated serious adverse event was grade 3 subcutaneous abscess that lasted 15 days, required hospitalization, and the patient recovered but discontinued rilzabrutinib after 84 days of treatment. The third patient had grade 2 gastritis and grade 2 gastroesophageal reflux disease on study day 136. The patient with concurrent lack of response and treatment-related grade 1 diarrhea starting on day 1 that changed to grade 2 on day 80 and returned to grade 1 on day 132.

**Efficacy**

Subgroup analysis of the primary endpoint by baseline platelet count ≥15×10^9^/L had a higher primary endpoint response (58% for 7/12 patients) than patients with baseline platelet count <15×10^9^/L (14% for 2/14 patients; Figure S2). Although this subgroup comparison was statistically significant by using Fisher’s exact test (*P*=0.04), the analysis is exploratory and based on a small sample size with no adjustment for covariates and should be interpreted with caution.

**Safety**

Three patients had serious adverse events that were not related to rilzabrutinib treatment per the investigator’s assessments. The first patient had non-treatment emergent grade 4 thrombocytopenia that began on 6 days before rilzabrutinib was initiated, continued after the start of rilzabrutinib dosing, and resolved on day 7 with no change in rilzabrutinib dosing. The second patient had grade 3 subcutaneous abscess on day 84 due to the same underlying medical condition that worsened and led to discontinuation of rilzabrutinib. The third patient had concurrent grade 3 post-procedural hemorrhage and grade 3 syncope that began on day 123 of treatment resulting in dose interruption.

**Exploratory markers and health-related quality of life**

Exploratory evaluation of hemolysis marker haptoglobin and immunoglobulins were within normal ranges at baseline and week 25 following rilzabrutinib treatment (table S4, appendix). Thrombopoietin (TPO) was above normal at baseline (median 175 ng/L [IQR, 117–243]), decreased at week 25 (median 110 ng/L [IQR, 58–176]), showing a median change from baseline to week 25 of –59 ng/L (IQR, –118 to 11). There was no significant difference in TPO levels for patients who were receiving concomitant TPO-RA or no concomitant TPO-RA. In 14 patients receiving rilzabrutinib with concomitant TPO-RA vs 12 patients with no concomitant TPO-RA, baseline levels of TPO were a respective median of 200 ng/L (IQR, 84–1346) vs 149 ng/L (IQR, 16–387; *P*=0.21), and respective changes from baseline at week 25 in TPO levels were –58 ng/L (IQR, –1021 to 20) and –26 ng/L (IQR, –118 to 24; *P*=0.41).

Examination of health-related quality of life (HRQOL) was an exploratory endpoint using the EuroQol-5 Dimensions 5-Level (EQ-5D-5L) plus Visual Analog Scale (VAS) and ITP Patient Assessment Questionnaire (ITP-PAQ) that measured HRQOL with scores ranging from 0 worst to 100 best HRQOL. The EQ-VAS visual scale recorded patient’s self-rated health state from 0 worst to 100 best possible. Median EQ-VAS at baseline was 78 (range, 8-100) with a median change from baseline (n=26) to week 25 (n=15) of +7 (range, –5 to 77) that indicated an improvement in HRQOL.

ITP-PAQ is a 44-item ITP-specific patient assessment questionnaire with scoring that ranges from 0 worst to 100 best health status. Clinically meaningful changes from baseline were defined as a range from 8-12 points for symptoms, bother, psychological, overall QOL, social activity, women’s reproductive health (ie, menstrual symptoms and fertility), and a range from 10-15 points for fatigue and activity (table S5).^1,2^ In the current study, improvements in ITP-PAQ scores were observed early in the study (figure S5) and median changes from baseline to week 25 (figure S5B) showed the greatest, clinically meaningful improvement in women’s reproductive health with a 21-point increase (range, 0-38; IQR, 0-38; median baseline: 54), as well as for overall, fatigue/sleep, and activity HRQOL scales with ~13-point increases (range, –23 to 75; median baseline: 52, 53, and 50, respectively). Numerical improvements in psychological, symptoms, social activity, and bother-physical health domains with 4- to 8-point increases (range, –13 to 56; median baseline: 70, 60, 75, 69, respectively) were also observed. The fear and work domains had high scores at baseline (≥90) with minimal changes over time.

**Table S1: Predictive platelet response following rilzabrutinib treatment during the 24-week main treatment period**

| Platelet response criteria | Patients met response criteria? | Positive predictive value | Negative predictive value |
| --- | --- | --- | --- |
| ≥50×10^9^/L at any time over the first 2 weeks | Yes n=10  No n=16 | 8/10 (80%)  — | —  15/16 (94%) |
| ≥50×10^9^/L at any time over the first 8 weeks | Yes n=12  No n=14 | 8/12 (67%)  — | —  13/14 (93%) |
| ≥30×10^9^/L and ≥20×10^9^/L above baseline over the first 2 weeks | Yes n=13  No n=13 | 8/13 (62%)  — | —  12/13 (92%) |

Data are n/n meeting (yes) or not meeting (no) platelet response criteria and the predictive percentage of patient’s responses positively or negatively. Positive predictive value is the probability of a patient meeting the primary endpoint given they had early platelet response as per criteria. Negative predictive value is the probability that a patient did not meet the primary endpoint given they did not have early platelet response as per criteria.

**Table S2: IBLS bleeding scale scores per location at the end of the main treatment period**

| IBLS Score | Baseline (N=26) | | | Week 25 (n=14) | | |
| --- | --- | --- | --- | --- | --- | --- |
|  | **0** | **1** | **2** | **0** | **1** | **2** |
| Skin history | 8 | 11 | 7 | 11 | 3 | 0 |
| Skin physical examination | 8 | 12 | 6 | 13 | 1 | 0 |
| Oral history | 18 | 5 | 3 | 13 | 1 | 0 |
| Oral physical examination | 21 | 4 | 1 | 13 | 1 | 0 |
| Epistaxis | 23 | 2 | 1 | 13 | 1 | 0 |
| Urinary | 26 | 0 | 0 | 13 | 1 | 0 |
| Intracranial | 25 | 1 | 0 | 14 | 0 | 0 |
| Gastrointestinal | 26 | 0 | 0 | 14 | 0 | 0 |
| Pulmonary | 26 | 0 | 0 | 14 | 0 | 0 |
| Subconjunctival hemorrhage | 26 | 0 | 0 | 14 | 0 | 0 |
| Gynecologic ^a^ | 9 | 1 | 0 | 3 | 0 | 0 |

Data are number of patients at each site per IBLS score (0-2).
^a^ For gynecologic IBLS scores, baseline n=10; week 25 n=3. IBLS=ITP Bleeding Scale.

**Table S3: All adverse events by maximum grade during the long-term extension period (n=11)**

|  | Adverse events due to any cause | | | | Treatment-related adverse events | | | |
| --- | --- | --- | --- | --- | --- | --- | --- | --- |
|  | Any grade | Grade 1 | Grade 2 | Grade 3 ^a^ | Any grade | Grade 1 | Grade 2 | Grade 3 |
| Any adverse events | 8 (73%) | 7 (64%) | 6 (55%) | 1 (9%) | 1 (9%) | 1 (9%) | 0 | 0 |
| Diarrhea | 1 (9%) | 1 (9%) | 0 | 0 | 1 (9%) | 1 (9%) | 0 | 0 |
| Nausea | 2 (18) | 2 (18%) | 0 | 0 | 0 | 0 | 0 | 0 |
| Cholelithiasis | 1 (9%) | 0 | 0 | 1 (9%) | 0 | 0 | 0 | 0 |
| Gamma-glutamyltransferase increased | 1 (9%) | 0 | 0 | 1 (9%) | 0 | 0 | 0 | 0 |
| Blood bilirubin increased | 1 (9%) | 0 | 1 (9%) | 0 | 0 | 0 | 0 | 0 |
| Bradycardia | 1 (9%) | 0 | 1 (9%) | 0 | 0 | 0 | 0 | 0 |
| Costochondritis | 1 (9%) | 0 | 1 (9%) | 0 | 0 | 0 | 0 | 0 |
| Hypertension | 1 (9%) | 0 | 1 (9%) | 0 | 0 | 0 | 0 | 0 |
| Transient ischemic attack | 1 (9%) | 0 | 1 (9%) | 0 | 0 | 0 | 0 | 0 |
| Vertigo positional | 1 (9%) | 0 | 1 (9%) | 0 | 0 | 0 | 0 | 0 |
| Alanine aminotransferase increased | 1 (9%) | 1 (9%) | 0 | 0 | 0 | 0 | 0 | 0 |
| Amnesia | 1 (9%) | 1 (9%) | 0 | 0 | 0 | 0 | 0 | 0 |
| Arthralgia | 1 (9%) | 1 (9%) | 0 | 0 | 0 | 0 | 0 | 0 |
| Aspartate aminotransferase increased | 1 (9%) | 1 (9%) | 0 | 0 | 0 | 0 | 0 | 0 |
| Back pain | 1 (9%) | 1 (9%) | 0 | 0 | 0 | 0 | 0 | 0 |
| Blood alkaline phosphatase increased | 1 (9%) | 1 (9%) | 0 | 0 | 0 | 0 | 0 | 0 |
| Butterfly rash | 1 (9%) | 1 (9%) | 0 | 0 | 0 | 0 | 0 | 0 |
| Erythema | 1 (9%) | 1 (9%) | 0 | 0 | 0 | 0 | 0 | 0 |
| Eyelid ptosis | 1 (9%) | 1 (9%) | 0 | 0 | 0 | 0 | 0 | 0 |
| Flank pain | 1 (9%) | 1 (9%) | 0 | 0 | 0 | 0 | 0 | 0 |
| Headache | 1 (9%) | 1 (9%) | 0 | 0 | 0 | 0 | 0 | 0 |
| Influenza-like illness | 1 (9%) | 1 (9%) | 0 | 0 | 0 | 0 | 0 | 0 |
| Malaise | 1 (9%) | 1 (9%) | 0 | 0 | 0 | 0 | 0 | 0 |
| Oropharyngeal pain | 1 (9%) | 1 (9%) | 0 | 0 | 0 | 0 | 0 | 0 |
| Palpitations | 1 (9%) | 1 (9%) | 0 | 0 | 0 | 0 | 0 | 0 |
| Paresthesia | 1 (9%) | 1 (9%) | 0 | 0 | 0 | 0 | 0 | 0 |
| Pruritus | 1 (9%) | 1 (9%) | 0 | 0 | 0 | 0 | 0 | 0 |
| Psoriasis | 1 (9%) | 1 (9%) | 0 | 0 | 0 | 0 | 0 | 0 |
| Pyrexia | 1 (9%) | 1 (9%) | 0 | 0 | 0 | 0 | 0 | 0 |
| Rash maculo-papular | 1 (9%) | 1 (9%) | 0 | 0 | 0 | 0 | 0 | 0 |
| Skin discoloration | 1 (9%) | 1 (9%) | 0 | 0 | 0 | 0 | 0 | 0 |
| Bleeding events |  |  |  |  |  |  |  |  |
| Epistaxis | 2 (18) | 1 (9%) | 1 (9%) | 0 | 0 | 0 | 0 | 0 |
| Hemorrhage | 1 (9%) | 1 (9%) | 0 | 0 | 0 | 0 | 0 | 0 |
| Infections and infestations |  |  |  |  |  |  |  |  |
| Upper respiratory tract infection | 1 (9%) | 0 | 1 (9%) | 0 | 0 | 0 | 0 | 0 |
| Varicella zoster virus infection | 1 (9%) | 0 | 1 (9%) | 0 | 0 | 0 | 0 | 0 |
| COVID-19 | 1 (9%) | 1 (9%) | 0 | 0 | 0 | 0 | 0 | 0 |

Adverse event data are shown by system organ class and preferred term for the number of patients (% of patients).
^a^ Included 1 patient with grade 3 cholelithiasis and grade 2/3 gamma-glutamyl transferase increase.

**Table S4: Effect of rilzabrutinib on markers of hemolysis (haptoglobin), immunoglobulin G (IgG), IgG1, IgG4, IgM, IgE, and thrombopoietin (TPO) levels during the main treatment period**

|  | Normal reference values | Baseline | Week 25 | Change from baseline to week 25 |
| --- | --- | --- | --- | --- |
| Haptoglobin, g/L | 0.3–2 | 0.75 (0.43–1.20)  (n=25) | 1.11 (0.08–1.41)  (n=10) | 0.33 (0.11–0.43) |
| IgG, g/L | 7–16 | 10.9 (9.2–14.0)  (n=26) | 9.2 (7.9–12.9)  (n=10) | –1.6 (–1.7 to 0.9) |
| IgG1, g/L | 4–10 | 7.5 (6.0–9.0)  (n=26) | 5.5 (5.0–9.0)  (n=10) | 0 (–1.0 to 0.0) |
| IgG4, g/L | 0–2 | 0 (0–1)  (n=26) | 0 (0–0)  (n=10) | 0 (0–0) |
| IgM, g/L | 0.4–2.3 | 1.0 (0.5–1.4)  (n=26) | 0.6 (0.4–1.2)  (n=10) | –0.2 (–0.4 to –0.1) |
| IgE, IU/L | 0–99 | 24 (9–72)  (n=26) | 28 (9–94)  (n=10) | 0 (0 to 6) |
| TPO, ng/L | 39–139 | 175 (117–243)  (n=26) | 110 (58–176)  (n=15) | –59 (–118 to 11) |

Data are median (IQR). Includes values for patients with data available from central laboratory analyses for the parameter assessed at baseline and post-baseline. IQR=interquartile range.

**Table S5: ITP-PAQ domain scores for the effect of rilzabrutinib on HRQOL**

| HRQOL Domains | Baseline (n=22) | Week 25 (n=14) | Change from baseline to week 25 (n=14) |
| --- | --- | --- | --- |
| Women’s reproductive health ^a^ | 54 (46–79) | 100 (100–100) | 21 (0–38) ^b^ |
| Overall HRQOL | 52 (30–67) | 82 (60–100) | 13 (7–23) ^b^ |
| Fatigue/sleep | 53 (38–75) | 84 (69–94) | 13 (6–19) ^b^ |
| Activity | 50 (13–75) | 75 (75–100) | 13 (0–25) ^b^ |
| Psychological health | 70 (50–85) | 90 (75–100) | 7.5 (0–10) |
| Social activity | 75 (44–88) | 94 (75–100) | 6 (0–13) |
| Symptoms | 60 (38–83) | 88 (71–100) | 6 (4-13) |
| Bother-physical health | 69 (44–83) | 89 (78–100) | 4 (–3 to 19) |
| Fear | 90 (75–95) | 100 (90–100) | 3 (0–5) |
| Work | 91 (50–100) | 100 (100–100) | 0 (0–6) |

Data are median (IQR) for ITP-PAQ scores.
Abbreviations: HRQOL, health-related quality of life; IQR, interquartile range; ITP-PAQ, ITP Patient Assessment Questionnaire.
^a^ Number of patients eligible for assessment (female and non-menopausal) for women’s reproductive health at baseline was n=9 and at week 25 was n=3.
^b^ Signifies minimum important differences representing clinically meaningful changes as defined by 8- to 12-point changes for symptoms, bother-physical health, psychological health, overall HRQOL, social activity, and women’s reproductive health and 10- to 15-point changes for fatigue and activity.^1,2^

**Study site investigators**

**Table S6: LUNA 2 study investigators and site locations**

| Study investigator names ^a^ | Study site location |
| --- | --- |
| Robert Bird | Princess Alexandra Hospital, Woolloongabba, Australia |
| Ralph Boccia | Center for Cancer and Blood Disorders, Bethesda, MD, USA |
| Lachezar H. Bogdanov | Clinic of Hematology, University Hospital, Pleven, Bulgaria |
| Nichola Cooper | Hammersmith Hospital, London, United Kingdom |
| Ilona Cunningham | Concord Repatriation General Hospital, Concord, Australia |
| Mamta Garg | Leicester Royal Infirmary, Leicester, United Kingdom |
| Terry B. Gernsheimer | University of Washington and Fred Hutchinson Cancer Center, Seattle, WA, USA |
| Isaac Goncalves | Royal Melbourne Hospital and Peter MacCallum Cancer Centre, Parkville, Australia |
| A. J. Gerard Jansen | Erasmus MC, University Medical Center, Rotterdam, the Netherlands |
| Zane Kaplan | Monash Medical Centre, Clayton, Australia |
| Milan Košťál | Fourth Department of Internal Medicine and Hematology, Faculty of Medicine, University Hospital of Hradec Králové, Hradec Králové, Czech Republic |
| David J. Kuter | Hematology Division, Massachusetts General Hospital, Harvard Medical School, Boston, MA, USA |
| Darla Liles | Pitt County Memorial Hospital, Greenville, NC, USA |
| Jiří Mayer | Masaryk University Hospital, Brno, Czech Republic |
| Vickie McDonald | Barts Health NHS Trust, The Royal London Hospital, London, United Kingdom |
| Charles Percy | Queen Elizabeth Hospital, Birmingham, United Kingdom |
| Michelle Sholzberg | St. Michael’s Hospital, Li Ka Shing Knowledge Institute, University of Toronto, Toronto, Ontario, Canada |
| Michael D. Tarantino | The Bleeding and Clotting Disorders Institute, University of Illinois College of Medicine-Peoria, Peoria, IL, USA |
| Paula F. Ypma | Department of Hematology, HagaZiekenhuis, Den Haag, the Netherlands |

^a^ Includes investigators who screened and/or enrolled patients; investigator names are listed alphabetically by last name.

**Figure S1: CONSORT diagram**^a^ One patient discontinued the study due to non–treatment-related, pre-existing grade 1 urinary tract infection, grade 3 hemorrhage, and grade 3 back pain; this patient received rilzabrutinib for 7 days.
^b^ One patient met exclusion criteria for hepatitis C antibody positive at screening, was erroneously enrolled, and exposed to rilzabrutinib for 21 days.
^c^ Two patients discontinued the study due to non–treatment-related grade 3 skin abscess (n=1) and grade 2 gastritis and gastroesophageal reflux disease (n=1).
^d^ One patient discontinued the study due to lack of response and treatment-related grade 2 diarrhea.

**Figure S2: Subgroup analysis of platelet response by baseline factors and concomitant therapy. Responses to prior therapy were defined as achieving platelet counts ≥50×10^9^/L on the prior therapy. *Note: Prior therapy consisted of n=0 TPO-RA only, n=4 CS only, and n=22 received both CS and TPO-RA; all patients were exposed to prior CS.
Abbreviations: CS, corticosteroids; ITP, immune thrombocytopenia; IVIg, intravenous immunoglobulin; TPO-RA, thrombopoietin receptor agonist.**

**Figure S3: Median (IQR) platelet counts over time with rilzabrutinib monotherapy or rilzabrutinib plus concomitant ITP therapy during the main treatment period. Abbreviations: IQR, interquartile range; ITP, immune thrombocytopenia.**
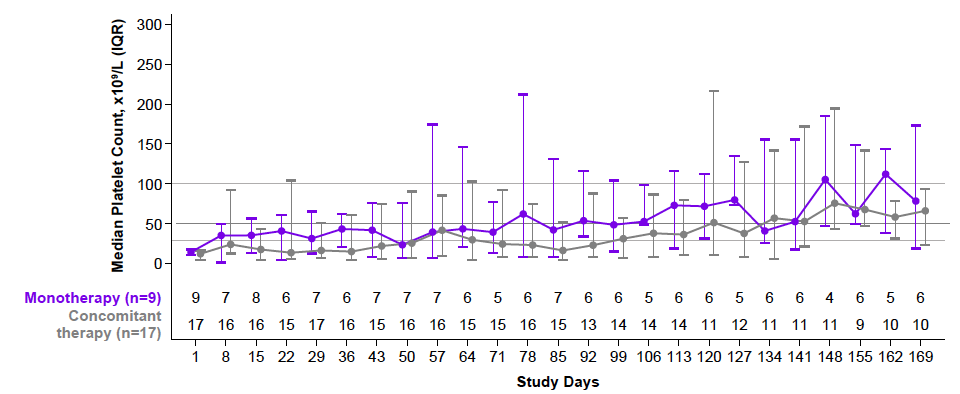


**Figure S4: Time to first platelet count ≥50**×**10^9^/L**

|  | Median time to first platelet count of ≥50×10^9^/L, days (IQR [95% CI]) |
| --- | --- |
| For all patients (n=26) | 95.5 (10–177 [15 to NA]) |
| For patients who achieved platelet count ≥50×10^9^/L (n=16) | 15 (8–75 [7–134]) |

Abbreviations: CI, confidence interval; IQR, interquartile range; NA, not applicable.

**Figure S5: Clinically significant and numerical changes in ITP-PAQ domain scores over time (A) and change from baseline to week 25 (B) for the effect of rilzabrutinib on HRQOL**

A.

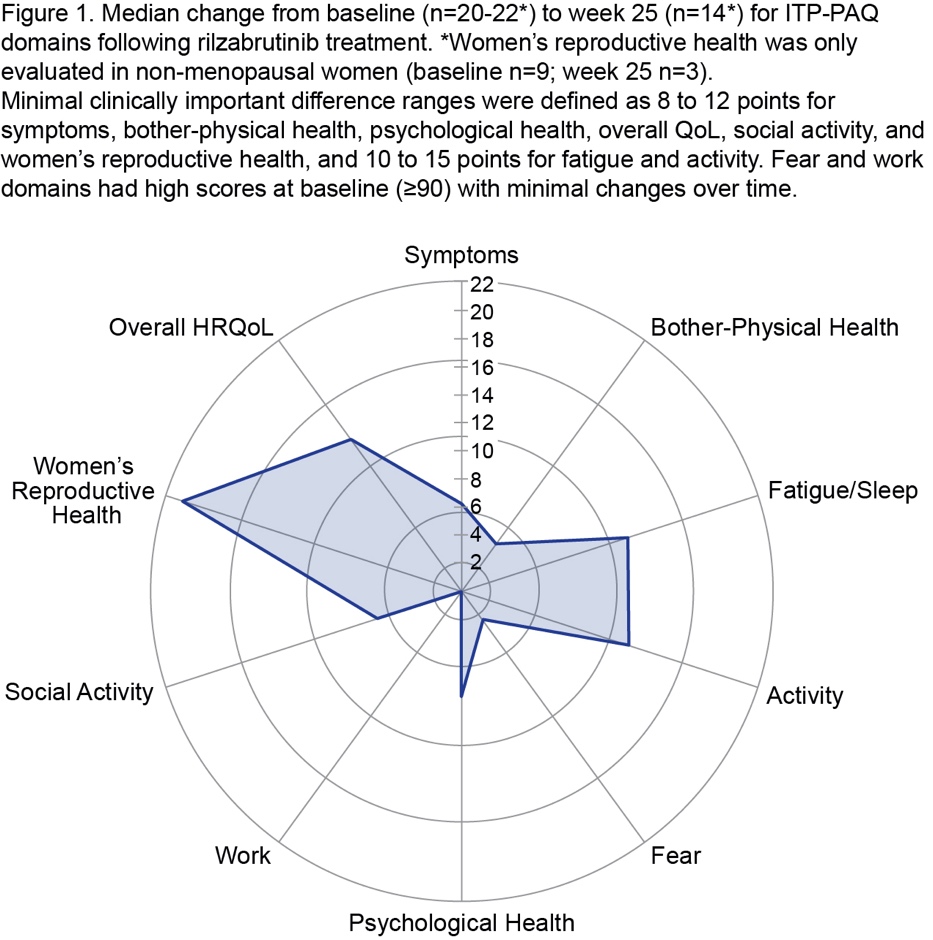
B.

**Supplemental References**

1. Mathias SD, Bussel JB, George JN, McMillan R, Okano GJ, Nichol JL. A disease-specific measure of health-related quality of life for use in adults with immune thrombocytopenic purpura: its development and validation. *Health Qual Life Outcomes.* 2007;5:11.

2. Mathias SD, Gao SK, Rutstein M, Snyder CF, Wu AW, Cella D. Evaluating clinically meaningful change on the ITP-PAQ: preliminary estimates of minimal important differences. *Curr Med Res Opin.* 2009;25(2):375-383.
